# The role of spatial accuracy and precision in hermit crab contests

**DOI:** 10.1016/j.anbehav.2020.07.013

**Published:** 2020-09

**Authors:** Sarah M. Lane, Mark Briffa

**Affiliations:** School of Biological and Marine Sciences, Animal Behaviour Research Group, University of Plymouth, Plymouth, U.K.

**Keywords:** accuracy, contests, repeated signals, resource-holding potential, skill, vigour

## Abstract

Understanding the determinants of fighting ability (or resource-holding potential, RHP) is key to elucidating the evolution of aggressive behaviour, as current tests of contest theory rely on realistic proxies for overall RHP. Traditionally, RHP is considered equivalent to body size but it is increasingly clear that a wider range of morphological and physiological traits contribute to fighting ability. In situations analogous to contests, such as courtship displays in animals and competitive sport in humans, the role of skill has long been appreciated but this component has been neglected in analyses of animal fights. Here, we investigated two spatial components of skill, accuracy and precision, during shell fights in hermit crabs, where an attacker repeatedly strikes (raps) its shell against that of a defender. By analysing the points of impact of these strikes, we found that attackers that rapped with coarse-scale accuracy were more likely to win the fight, indicating that the ability to target a ‘sweet spot’ on the defender's shell is an important determinant of contest success. Furthermore, we found that this element of skill correlated with temporal performance (vigour). Taken together these results show that spatial skill is an RHP component. Moreover, in contrast to the traditional assumption that fighting ability is equivalent to body size, RHP is actually underpinned by a suite of interlinked traits including performance capacities, morphology and skill.

## Highlights

• Fighting often involves the repeated performance of agonistic behaviours.
• Individuals may vary in how skilfully they perform such behaviours.
• We investigate the link between spatial skill and fighting success in hermit crabs.
• We find that winners perform rapping behaviour more skilfully than losers.
• Our results indicate that skill is tightly correlated with other fighting traits.

To maximize fitness, individuals often have to fight conspecifics for ownership of critical resources. Access to food, shelter, mates and ownership of territories containing these resources is dependent on winning fights, so considerable effort has gone into modelling the evolution of aggressive behaviour. The concept of resource-holding potential (RHP), or fighting ability (Parker, 1974), is central to our understanding of animal contests because we rely on measures of fighting ability to distinguish between different explanations for the functions of agonistic behaviour (Arnott & Elwood, 2009; Briffa & Elwood, 2009; Chapin, Peixoto, & Briffa, 2019; Palaoro & Briffa, 2016). While relevant measures of RHP are of critical importance, a range of different traits may contribute to an individual's ability to win fights. RHP is typically attributed to morphological traits that are assumed to give an individual a physical advantage (e.g. body size). However, escalated fights are known to occur more often between similarly sized individuals in the wild (Morrell, Backwell, & Metcalfe, 2005), indicating that traits other than body size might differentiate winners and losers outside of a laboratory setting (e.g. winner and loser effects, Hsu, Earley, & Wolf, 2006; resource ownership, Kasumovic, Elias, Sivalinghem, Mason, & Andrade, 2010; see Vieira & Peixoto, 2013 for a review of RHP traits). Recent studies have begun to explore the role of performance traits in determining RHP and thus contest success. Unlike static morphological traits, performance traits capture the dynamic behaviour of individuals during fights. Fighting performance is generally thought of in terms of vigour, the rate at which an individual carries out a repeated agonistic behaviour (Byers, Hebets, & Podos, 2010). Vigour is known to be an important determinant of contest outcome for various species including the telson sparring matches of the mantis shrimp, *Neogonodactylus bredini* (Green & Patek, 2015) and the shell fights of the European hermit crab, *Pagurus bernhardus* (Briffa, Elwood, & Dick, 1998). However, fighting performance can also vary in terms of skill. Skill is defined as the ability to perform a challenging behaviour well (Byers et al., 2010) incorporating efficiency, accuracy, precision and appropriateness (see Briffa & Lane, 2017 for a review).

Skill in the context of animal behaviour has thus far mostly been studied with respect to elaborate courtship displays. Byers et al. (2010) proposed that motor performance during such displays should provide females with more reliable information about a male's underlying genetic quality than his sexual ornaments, as motor performance reflects aspects of whole organism performance linked to survival. Indeed, studies of multimodal courtship displays in tropical birds have since found that not only do males differ in the vigour and skill with which they perform displays (Manica, Macedo, Graves, & Podos, 2017) but also for some species (specifically, golden-collared manakins, *Manacus vitellinus*), female preference, and therefore mating success, is based on these differences (Barske, Schlinger, Wikelski, & Fusani, 2011). Despite striking similarities between the complexity and repetition of behaviours exhibited during courtship and contests (Briffa & Lane, 2017; Mowles & Ord, 2012), the importance of skill for success in animal contests has yet to be examined in detail, with data currently restricted to a single study on hermit crabs. This study found that during contests over gastropod shells, in which the attacker hits its shell against that of the defender (a behaviour termed ‘rapping’), the efficiency with which the attacker rapped (measured as the distance the attacker displaced its shell during rapping) was more important in determining contest outcome than the relative size difference of opponents (Briffa & Fortescue, 2017). Similarly, within the field of sports science, skill can be more important than athleticism in determining victory in competitive sports (Wilson et al., 2017). Therefore, in the case of animal contests, measures of skill could represent a component of fighting ability that has thus far been overlooked. Moreover, by failing to capture major components of fighting ability we reduce our capacity to test the large body of theory concerning the evolution of fighting, which requires realistic proxies of RHP.

Typically, to win a fight, an opponent must convince its rival to give up. However, the agonistic behaviour required to elicit this giving up decision is costly in terms of time and energy. Therefore, fighting in an efficient way will optimize the benefits of winning, i.e. allowing victory to be achieved with the minimal possible outlay in terms of time and energy costs, henceforth referred to as ‘effort’ (Briffa & Elwood, 2004). For fights in which opponents hit one another repeatedly, the spatial placement of blows may influence the likelihood and speed of success. For instance, it can be important for an individual to exhibit a high degree of accuracy, targeting a specific body area or ‘sweet spot’ in order to elicit the desired retreat from the recipient (e.g. telson sparring in mantis shrimp, Green & Patek, 2015). Furthermore, as most fights will not be won with a single blow, victory may require an individual to hit the same ‘sweet spot’ repeatedly, striking with a high degree of precision. However, the importance of these spatial components of skill have yet to be investigated in animal contests. The European hermit crab relies on empty gastropod shells to protect its soft abdomen, exhibiting preferences for both the species and size of shell, with optimal shell size changing relative to their growing body size (Elwood & Neil, 1992). Hermit crabs inhabiting a suboptimal shell will readily initiate fights with crabs that possess a more suitably sized shell. During fights, the crabs take on specific roles of attacker and defender. Once the fight begins, the defender withdraws tightly into its shell while the attacker grabs the defender and raps its shell vigorously against that of the defender. The attacker performs multiple bouts of rapping, in between which (during pauses in rapping) it uses its chelipeds to attempt to pull the defender out of its shell (Dowds & Elwood, 1983). However, an eviction is only possible if the defender releases its internal abdominal grasp on the shell. Although it is known that an attacker can increase its chances of securing an eviction by rapping vigorously (Briffa et al., 1998, 2003; Elwood, Wood, Gallagher, & Dick, 1998), why shell rapping causes defenders to release their internal grasp is still unclear. At present there are two hypotheses for this response: (1) rapping dislodges the defender's grasp by targeting an internal anchor point and (2) rapping causes a general reduction in tenacity across the defender's abdominal musculature due to a general reflex contraction known to occur in response to vibration (Chapple, 1993). In addition, there is evidence that (3) rapping contains information about the attacker's likely persistence (a component of RHP), which the defender can access. This final possibility is not mutually exclusive of either hypothesis 1 or 2 outlined above. Rather, it appears that although rapping does inflict direct costs on defenders, which drive outcomes in a proportion of fights, defenders that receive highly vigorous rapping might choose to give up early, before they experience the full impact of these costs (Briffa & Elwood, 2004). Nevertheless, the origin of those direct effects on defenders remains unknown. Hypotheses 1 and 2 present predictions for the spatial distribution of raps. If the first hypothesis is correct, we would expect successful attackers to show a high degree of accuracy and precision, concentrating raps on a certain area of the defender's shell, a ‘sweet spot’. If the second hypothesis is correct, however, we would expect successful attackers to distribute their raps widely across the surface of the defender's shell, potentially still exhibiting a high degree of accuracy, but importantly showing lower precision and greater variance in rap position compared with unsuccessful attackers. Alternatively, if vigour is the sole determinant of a rapper's success (as is one hypothesis associated with point 3), then we would expect to find no effect of accuracy or precision on fight outcome. Here we used *P. bernhardus* to investigate these hypotheses and, more broadly, to examine the relative importance of temporal (vigour) and spatial (skill) performance components in determining contest outcome. As there are no data to suggest where a ‘sweet spot’ might be located, we analysed both the coarse- and fine-scale spatial distributions of the points of impact (POIs) of repeated raps in an effort to ascertain whether accuracy and precision are important at a local or general level: for example, is it enough to hit a certain part of the shell (e.g. body whorl, apex, lip) or must the attacker hit more localized targets to secure victory?

## Methods

### Crab Collection and Staging Fights

*Pagurus bernhardus* were collected from Hannafore Point, Looe (Cornwall, U.K.; Grid reference: SX 255523) between February and June 2019. They were returned to the laboratory within 1–2 h of collection and kept in groups of 70–100 individuals in 80-litre tanks containing aerated sea water. They were maintained at 15 °C on a 12:12 h light:dark lighting cycle and fed ad libitum on frozen white fish once a week.

After at least a 24 h acclimation period, crabs were carefully removed from their gastropod shells using a bench vice. Crushing the original shell in a vice allows us to quickly remove the crab from that shell without causing any damage to the crab itself. Excess sea water was removed from the crabs using absorbent paper. Crabs were then weighed (Weight range 0.2–1.73 g, *N* = 248) and allocated to pairs consisting of a larger crab (the potential attacker) and a smaller crab (the potential defender). Only male crabs without any missing appendages or visible parasites were used; all other individuals were given a new shell and returned to the field site. Within pairs, the attacker received a shell that was 60% of its optimal shell weight while the defender's shell was 100% of the optimal weight for the attacker. Preferred shell weight was calculated from regression equations derived from a previous shell selection experiment (Briffa & Elwood, 2007). To identify points of impact (POI) from repeated shell rapping on the defender's shell, a blue grease pencil (Dixon Phano china marker) was used to coat the body whorl of the shell given to the attacker. Once provided with new shells, the crabs were placed individually in plastic dishes (12 cm diameter) containing aerated sea water and left to acclimatize to their new shell for 15–20 h. After this time, the defender was introduced into the dish containing the attacker and the pair was observed for 20 min. During this time, if a fight occurred, the temporal pattern of rapping (total number of raps and bouts, mean number of raps per bout) was recorded by the observer using JWatcher V.1.0 (Blumstein & Daniel, 2007) along with the outcome of the fight (eviction or noneviction). If an eviction occurred, the exchange of shells was quickly intercepted by the observer to obtain the defender's shell. If a noneviction occurred (i.e. the attacker gave up), the defender was placed back in its original plastic dish and provided with a shell of 100% optimal weight to provoke the defender to move into this new shell. A defender would typically move into this new shell within 1 h of the fight ending. We found no evidence of grease pencil marks being left behind on the plastic surface of the dish and were thus confident that rap marks remained unchanged on the shell during this time. The shell occupied by the defender during the fight was then retained to measure the POIs of raps (see below). All fights were recorded using a Canon LEGRIA HF R706 High Definition Camcorder (Canon, Tokyo, Japan). A total of 124 interactions were observed resulting in 94 fights with 80 ending in an eviction and 14 ending in a noneviction.

### Measuring Points of Impact

**Figure 1:**
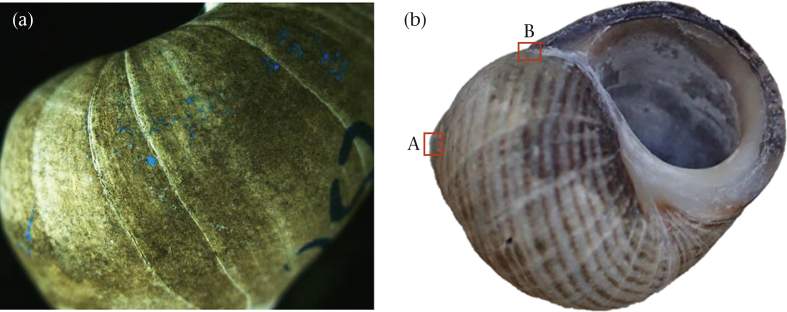
Measuring the spatial distribution of raps. (a) Shell showing points of impact (POIs) left behind by attacker in blue grease pencil. (b) Fine-scale distribution: distance measured from POIs to shell landmarks A and B.

**Figure A1:**
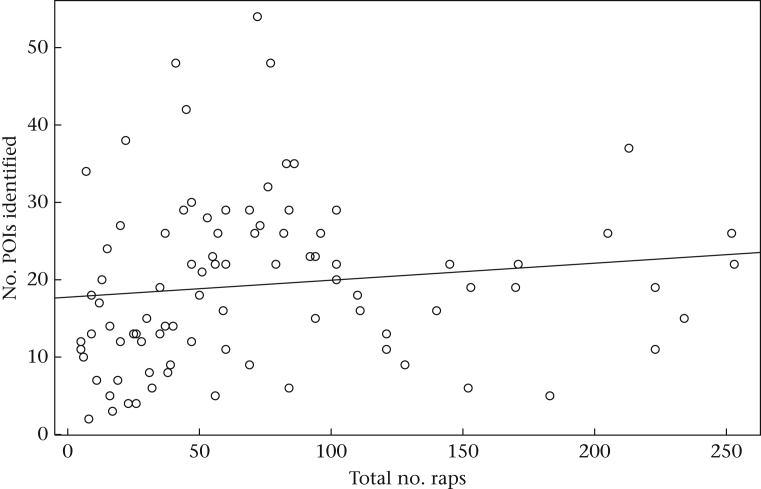
Correlation between number of points of impact (POIs) identified and the total number of raps performed.

After the fight, the defender's shell was photographed using a Leica M206C microscope (Leica Microsystems Ltd, Heerbrugg, Switzerland) equipped with a QImaging Retiga R6 camera (Cairn Research Ltd, Faversham, Kent, U.K.) connected to a computer. The shell was photographed to capture the positions of all marks of blue grease pencil (POIs) left behind by the attacker's raps (Fig. 1). To make the photographed POIs more visible by eye, these marks were then overlaid with a Sharpie pen. To assess whether this protocol provided an accurate representation of raps performed by the attacker, we analysed the relationship between the number of POIs identified and the total number of raps recorded for individual fights. The number of POIs was significantly positively correlated with the total number of raps performed (*r*_*τ*_ = 0.17, *P* = 0.018); however, the number of POIs identified was generally not equal to the number of raps recorded suggesting that while some attackers left multiple marks behind per rap, others performed far more raps than was discernible from the POIs (especially attackers that performed more than 100 raps; see Appendix Fig. A1). Thus, individual POIs were not considered representative of individual raps but rather we used the overall pattern of POIs as a proxy for the spread of raps performed across the fight.

**Figure 2:**
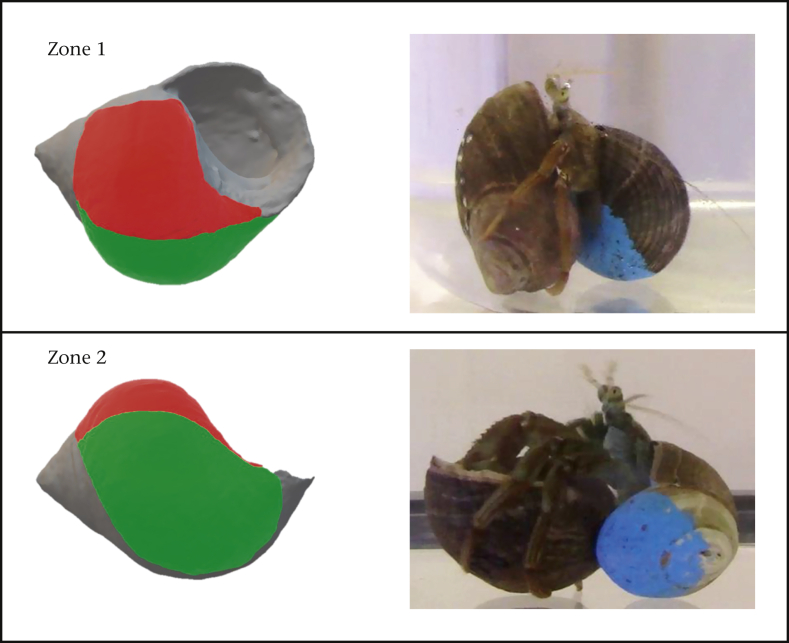
Coarse-scale distribution of raps was measured as the proportion of points of impact that fell in two zones, zone 1 (red) and zone 2 (green), which relate to the target areas available to attackers when rapping in the two most common positions as seen on the right.

To measure the fine-scale spatial distribution of raps, the observer measured the distance from each POI to two landmarks on the shell: (1) the apex (landmark A) and (2) the point at which the outer lip meets the body whorl (landmark B; Fig. 1). Attackers generally hold the defender's shell in one of two positions during a fight, with the apertures of both shells either parallel or perpendicular to each other (Fig. 2). To measure the coarse-scale distribution of raps, we thus divided the defender's shell into two zones which represent the target areas available for rapping by attackers in these two positions: zone 1 was the body whorl adjacent to the upward-facing aperture (average proportion of POIs landing in zone 1 = 0.57) while zone 2 was the body whorl perpendicular to the aperture (average proportion of POIs landing in zone 2 = 0.38). As can be seen from the summed proportion of POIs found in these zones, zones 1 and 2 jointly received 95% of all raps. The proportion of POIs within each zone was then calculated from the total number of POIs on the shell. All POI measurements were made blind by the same researcher.

### Ethical Note

The research described in this study adheres to the ASAB Guidelines for the Use of Animals in Research. After use in this study all hermit crabs were returned to the collection site at Hannafore Point. No licences or permits were required for this study.

### Statistical Analyses

#### Coarse-scale spatial distribution of raps

We analysed the effect of proportion of raps within zone 1 and zone 2 (and their interaction) on contest outcome (eviction or noneviction) using a generalized linear model (GLM) with binomial error family. We controlled for the effect of size by including relative weight difference calculated as RWD = 1–(defender weight/attacker weight) (as calculated in Briffa et al., 1998) in both analyses as a covariate.

Subsequent to analysis of the relation between coarse-scale placement and chance of victory, correlations between spatial (proportion of raps in zone 1) and temporal (total number of raps and average number of raps per bout, respectively) rap parameters were analysed using Kendall's rank correlation (tau) due to the non-normal nature of the spatial parameter and the presence of tied ranks. On inspection of the data, three outliers were identified. The following analysis was run twice, once with and once without the outliers to test whether their presence or absence influenced the results. The outliers made little difference to the results but made the figures less clear and thus the findings reported below are based on the analysis with these outliers removed (details of the analysis containing the outliers is available in the Supplementary material).

#### Fine-scale spatial distribution of raps

Before analysing the fine-scale distribution of POIs, we scaled POI measures for defender shell size by dividing each POI measure by the cube root of the defender's shell weight. This scaling allowed us to account for the effect of defender shell size on POI measurements. These scaled POI measures were then used for the following analyses.

To analyse fine-scale POI distribution, we used double hierarchical GLMs (DHGLMs) which comprise a ‘mean model’ and a ‘standard deviation (SD) model’. By running these models we could simultaneously analyse the fixed effects and random effects on (1) mean POI positions relative to landmarks A and B (separate analyses were conducted with distance to A and distance to B as the response variable, respectively), our measure of accuracy and (2) the variance around those positions, our measure of precision. The fixed effects were contest outcome (eviction or noneviction), average number of raps per bout (vigour) and relative weight difference (RWD). Individual ID was included as a random effect in both the ‘mean model’ and ‘SD model’ to account for the fact that multiple POI positions were measured for each individual. These analyses were carried out using JAGS software (Plummer, 2003) in conjunction with the RJAGS (Plummer, 2014) R package (R Studio Team, 2015). Since these DHGLMs are analysed within a Bayesian framework the primary means of assessing the importance of an effect is by inspecting 95% credible intervals (CIs) for overlap with zero. However, following Bridger, Bonner, and Briffa (2015) we have supplemented this information by calculating pseudo *P* values (henceforth ‘*P*’). See the Supplementary material for the relevant R code and for further details of the modelling conditions.

## Results

### Coarse-scale Spatial Distribution of Raps

**Figure 3:**
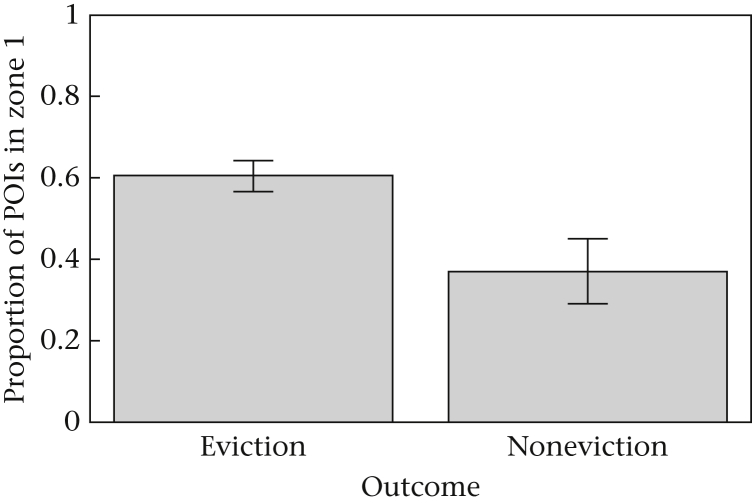
The effect of the proportion of points of impact (POIs) in zone 1 on the likelihood of eliciting an eviction.

**Figure 4:**
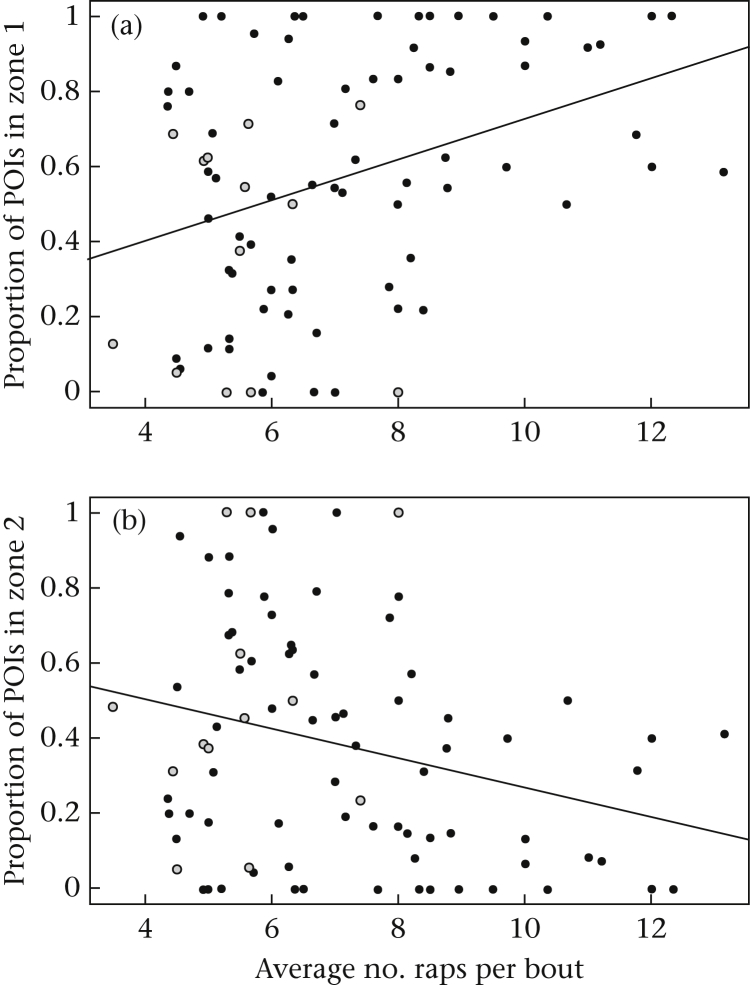
Correlations between average number of raps per bout and the proportion of points of impact (POIs) landing in (a) zone 1 and (b) zone 2. Black circles show data for evictions and grey circles show data for nonevictions.

There was a significant effect of the proportion of POIs in zone 1 (χ^2^_1, 87_ = 5.94, *P* = 0.015) but not zone 2 (χ^2^_1, 87_ = 0.45, *P* = 0.50) on fight outcome, indicating that attackers that successfully elicited an eviction landed a higher proportion of raps in zone 1 than attackers that failed to elicit an eviction (Fig. 3). There was also a significant positive correlation between the proportion of POIs in zone 1 and the average number of raps per bout (*r*_*τ*_ = 0.23, *P* = 0.002; Fig. 4a), whereas the proportion of POIs in zone 2 was negatively correlated with average raps per bout (*r*_*τ*_ = -0.167, *P* = 0.025; Fig. 4b). We found no significant correlations between the proportion of POIs in zone 1 (*r*_*τ*_ = 0.059, *P* = 0.422) or zone 2 (*r*_*τ*_ = 0.021, *P* = 0.78) and the total number of raps performed.

### Fine-Scale Spatial Distribution of Raps

**Figure 5:**
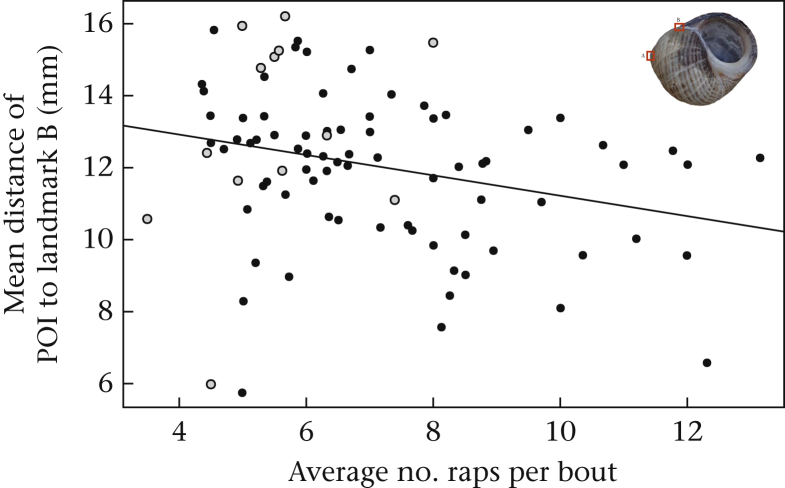
Relationship between average number of raps per bout and mean distance of points of impact (POIs) to landmark B. Inset shell shows location of landmarks. Black circles show data for evictions and grey circles show data for nonevictions.

When points of impact were analysed across the whole shell, there was no evidence for any relationships between fight outcome and either mean POI position or variance in POI positions, with respect to landmark A or landmark B (see Table 1). However, we did find a significant effect of the average number of raps per bout on the mean position of POIs relative to landmark B (γ = -0.06, 95% CIs = [-0.128, 0.001], *P* = 0.031; Fig. 5).

**Table 1:** Results of DHGLM analyses of fine-scale spatial distribution of points of impact across the whole shell

Effect	Estimate mean	Estimate SD	95% CI lower	95% CI upper	*P*
**Distance to landmark A**					
Mean model					
Relative weight difference	-0.041	0.070	-0.181	0.09	0.564
Outcome	0.095	0.214	-0.304	0.519	0.497
Average raps per bout	0.012	0.029	-0.048	0.067	0.710
SD model					
Relative weight difference	-0.169	0.350	-0.910	0.696	0.103
Outcome	-0.273	0.755	-1.537	1.552	0.517
Average raps per bout	1.132	1.486	-0.029	4.028	0.791
**Distance to landmark B**					
Mean model					
Relative weight difference	-0.079	0.070	-0.220	0.054	0.280
Outcome	-0.110	0.204	-0.505	0.309	0.716
Average raps per bout	-0.060	0.034	-0.128	0.001	**0.031**
SD model					
Relative weight difference	-0.034	0.046	-0.124	0.059	0.476
Outcome	0.244	0.147	-0.044	0.540	0.120
Average raps per bout	0.013	0.02	-0.026	0.053	0.496

CI: credible interval. Significant result (*P* < 0.05) is highlighted in bold.

Since the coarse-scale analysis indicated that hitting in zone 1 was important, we repeated the DHGLM analysis only for POIs that landed in zone 1, to determine whether accuracy and precision within this zone were related to outcome. We found no evidence for any statistically significant effects of outcome, average raps per bout or RWD on the mean or variance in position of POIs in zone 1 in relation to landmark A or B. See Table A1 in the Appendix for full results.

## Discussion

Fighting performance is generally thought of in terms of the rate of agonistic behaviour (vigour), but recent evidence suggests that the skill with which such behaviour is performed may be just as important in determining victory. Briffa and Fortescue (2017) demonstrated that efficient movement patterns increased the chance of victory in hermit crab fights and here we studied the spatial components of skill, accuracy and precision, by analysing the spatial distribution of raps (using POIs as a proxy) on the defender's shell. Our results indicate that successful attackers demonstrated a greater degree of coarse-scale accuracy and precision than unsuccessful attackers, concentrating a high proportion of raps on one particular area of the defender's shell, zone 1. Furthermore, the proportion of raps in zone 1 was positively correlated with average number of raps per bout, while the proportion of raps in zone 2 was negatively correlated with this temporal performance measure, indicating that skill correlates with vigour. Although we found no effect of fine-scale accuracy or precision on fighting success, a marginally significant association between number of raps per bout and the mean distance from landmark B (the point at which the outer lip meets the body whorl), suggests that fine-scale accuracy may be similarly correlated with vigour.

Hermit crab fights are asymmetrical, each crab taking on the role of either the attacker or the defender. It has long been known that an attacker can increase its chances of eliciting an eviction by rapping vigorously (Briffa et al., 1998), but exactly why shell rapping causes defenders to release their grip and give up their shell has remained unclear. The results of our study suggest that attackers are more likely to elicit an eviction when they focus their rapping efforts on a specific area of the defender's shell. This is consistent with the hypothesis that shell rapping dislodges the defender's internal grasp by targeting an underlying anchor point. Hermit crabs are thought to hold onto their shells using both their abdominal musculature and their uropods. At present, the exact position of the defender (and thus of these anchoring structures) within its shell during a fight has not been identified but from experience of cracking hermit crabs out of their shells, it is probable (S.M. Lane & M. Briffa, personal observation) that zone 1 is located directly above the defender's abdomen. The abdominal muscles of hermit crabs have previously been shown to respond to applied vibration by exhibiting bursts of reflex expansion and contraction (Chapple, 1993), suggesting not only that the abdomen may be sensitive to the effects of rapping, but that this muscular reaction could cause defenders to loosen their internal grasp on the shell. Whether rapping in zone 1 reduces the general tenacity of the abdominal muscles, the uropods specifically, or both, remains to be determined.

As well as coarse-scale accuracy being related to contest outcome, it also appears that skill and vigour (and potentially morphology) are interrelated. The proportion of POIs in zone 1 was positively correlated with the average number of raps per bout (a measure of vigour known to affect fighting success, Briffa et al., 1998), indicating that skilful attackers also performed raps with greater vigour. The negative correlation between proportion of raps in zone 2 and average raps per bout demonstrates that attackers that focused a high proportion of their raps in zone 2 (an area not associated with fighting success) had a low degree of vigour. This pattern is also reflected at the fine scale by a negative relationship between average raps per bout and mean distance to landmark B (where the outer lip meets the body whorl), with attackers that landed POIs further away from this landmark showing lower vigour. The straight-line trajectory from this landmark spreads across zone 1 and directly into zone 2; thus, the further away from landmark B, the more likely the POI is to have landed in zone 2 and vice versa. This correlation between spatial skill and vigour makes it difficult to disentangle their relative importance in determining contest success. One possibility, as discussed above, is that hitting in the right place is important for securing evictions because it relates to an underlying anchor point, which when struck repeatedly, reduces the ability of the defender to maintain its internal grip on the shell, using its abdominal musculature and terminal uropod. In this instance the correlation between skill and vigour would suggest that attackers skilled at hitting in the right place are also capable of carrying out more raps per bout, that is, some attackers are better all-round fighters. However, another possibility is that the spatial distribution of raps is not important for fight outcome per se, but rather that rapping in the right area enables attackers to perform more raps per bout which then in turn determines fighting success. This could also explain why we did not find any significant effect of fine-scale spatial distribution on fight outcome. The coarse-scale spatial distribution of raps is likely to be determined by the way in which an attacker holds the defender's shell, a factor that can change notably between the bouts of a single fight (S.M. Lane, personal observation). If an attacker does not have a stable grasp on the defender's shell, it is probable that it will (1) be more likely to slip while rapping and thus spread its raps more widely across the defender's shell and (2) be able to perform fewer raps before it must pause and reposition itself to gain greater traction.

Although we have focused on the behaviour of the attacker, it is also possible that defenders, although withdrawn into their shell for most of the fight, still perform intermittent defensive activities between bouts of rapping, potentially hampering the attacker's behaviour or indicating skilful defence in its own right. Because of their withdrawn posture, it is difficult to assess the behaviour of defenders during a fight. However, defenders are known to perform ‘cheliped flicking’ within the aperture along with occasional partial emergence and grappling with the attacker. Although cheliped flicking has not been found to be an important determinant of fighting success (M. Briffa, *unpublished data)*, the effect of the defender's behaviour on the ability of the attacker to rap skilfully warrants investigation. Furthermore, hermit crabs, like many animals, perform signalling displays before escalating to physical fights; thus, it is possible that attackers may assess the motor performance of defenders during this display (before they withdraw into their shells) to decide whether or not to engage in a fight. Byers et al. (2010) suggested similarly that females may be able to use male courtship displays to detect subtle differences in motor performance between males, informing their choice of a mate.

Although the study of skill in animal behaviour is in its infancy, it is already clear that the relationship between skill and other RHP traits (in particular, vigour) is complicated. For instance, while data from our current experiment and previous work on contests (Briffa & Fortescue, 2017) demonstrate a positive correlation between skill and vigour, data from the leaping courtship displays of blue-black grassquits, *Volatinia jacarina*, indicate the presence of a trade-off between skill and vigour (Manica et al., 2017). Manica et al. (2017) found a negative correlation between leap height (skill) and the number of leap displays performed (vigour), but only in smaller males, suggesting that the ability to maximize quality and quantity in these elaborate displays may be size dependent. Thus, the relationship between skill and vigour may vary between contexts and between individuals. Understanding the underlying factors that determine the direction of the relationship between skill and vigour will require skill to be studied across an extended range of examples, varying in the type of skill parameter and the complexity of behaviour measured (i.e. spatial accuracy versus motor movement, etc).

Skill is generally associated with the performance of difficult or complex tasks (Byers et al., 2010). Indeed, skill is important for complex courtship displays composed of a combination of motor and vocal elements (Manica et al., 2017), but our study indicates that skill is also important for the successful performance of relatively simple, but repeated, behaviours. This idea is further supported by studies of human combat sports such as boxing, where winners show a higher level of accuracy than losers, landing a greater proportion of on-target punches (Ashker, 2011). Repeated signals are commonplace in both contests and courtship, varying in cost and complexity (Mowles & Ord, 2012). The use of repeated signals in courtship has been suggested to play several roles (see Mowles & Ord, 2012 for a review), including indicating quality through the demonstration of sustained vigour (Byers et al., 2010). Our findings suggest that individuals may also vary in their ability to perform these repeated signals skilfully, with accuracy and precision. However, further investigation is required to determine whether the skill with which repeated signals are performed is important in influencing the decision making of signal recipients (e.g. the decision to give up in a contest, or the decision to mate).

### Conclusions

Here, we have demonstrated for the first time that spatial components of skill (accuracy and precision) contribute to the chance of victory in contests. In fighting hermit crabs, the ability to hit a specific area of the defender's shell repeatedly is clearly an important determinant of victory. Similar capacities could determine fight outcomes in other species and (alongside traditional morphological RHP measures and performance measures, e.g. vigour) such measures of spatial skill should be incorporated into our concepts of fighting ability in animals. In particular, it seems clear that skill contributes to RHP, and so it should be incorporated into analyses of contest behaviour that rely on realistic RHP measures.

## Conflict of Interest

No competing interests declared.

## Figures and Tables

**Table A1:** Results of DHGLM analyses of fine-scale spatial distribution of points of impact that landed in zone 1.

Effect	Estimate mean	Estimate SD	95% CI lower	95% CI upper	*P*
**Distance to landmark A**					
Mean model					
Relative weight difference	-0.036	0.091	-0.215	0.149	0.705
Outcome	0.012	0.256	-0.471	0.516	0.801
Average raps per bout	0.056	0.036	-0.018	0.123	0.133
SD model					
Relative weight difference	-0.360	0.773	-2.977	0.023	0.107
Outcome	-0.351	1.632	-5.646	0.604	0.489
Average raps per bout	0.218	0.556	-0.017	2.108	0.228
**Distance to landmark B**					
Mean model					
Relative weight difference	-0.122	0.085	-0.289	0.041	0.135
Outcome	-0.418	0.274	-0.932	0.108	0.113
Average raps per bout	-0.031	0.335	-0.097	0.034	0.342
SD model					
Relative weight difference	-0.034	0.158	-0.606	0.074	0.954
Outcome	-0.237	0.865	-4.008	0.207	0.895
Average raps per bout	0.063	0.258	-0.024	1.084	0.862

CI: credible interval.
