# Supplementary material for: The role of spatial accuracy and precision in hermit crab contests
Source: Anim Behav. 2020 Sep;167:111–8. doi: 10.1016/j.anbehav.2020.07.013 (PMC7487773; doi:10.1016/j.anbehav.2020.07.013)
Supplement: Multimedia component 1 [file mmc1.docx]

**Supplementary Material**

**HITTING THE SWEET SPOT: THE ROLE OF ACCURACY AND PRECISION IN HERMIT CRAB CONTESTS**

**Sarah M. Lane* & Mark Briffa**

School of Biological and Marine Sciences, Animal Behaviour Research Group, University of Plymouth, Plymouth, Devon, PL4 8AA, UK.

* Corresponding email: [sarah.lane@plymouth.ac.uk](mailto:sarah.lane@plymouth.ac.uk)

**Results of coarse-scale analysis with outliers (*N* = 90): -**

Correlation between proportion of raps in zones and average no. raps per bout: -

Zone 1: *r _τ_* = 0.23, *P* = 0.002

Zone 2: *r _τ_* = -0.18, *P* = 0.013

Correlation between proportion of raps in zones and total no. raps: -

Zone 1: *r _τ_* = 0.06, *P* = 0.39

Zone 2: *r _τ_* = 0.18, *P* = 0.80

**DHGLM R code**

**Part 1**

**#run this model for random slopes on treatment.**

##### Likelihood contribution #####

model{for(i in 1:n){ y[i] ~ dnorm(muy[i],tau[i]) #each y has its own mean and precision

tau[i] <- 1/sdy[i]/sdy[i] # express the model in terms of the standard deviation

## Model for the mean

muy[i] <- beta[1] +

beta[2]*StRWDx[i] +

beta[3]*Outx[i] +

beta[4]*AvgRaps[i] +

re.mu[IDx[i],1] #random interce #random slope across treatments

## Model for the st. dev

log(sdy[i]) <- gamma[1] +

gamma[2]*StRWDx[i] +

gamma[3]*Outx[i] +

gammae[4]*StAvgRaps[i] +

re.sd[IDx[i]] #random intercept}

## Redundant random effects model

redun.mu[1] ~ dnorm(0,1) # redundant parameterization of Gelman

redun.sd ~ dnorm(0,1)

for(i in 1:nID){

re.mu[i,1] <- redun.mu[1]*phi.mu[i,1]

re.sd[i] <- redun.sd * phi.sd[i]}

## Prior for beta, gamma

for(h in 1:4){

beta[h] ~ dnorm(0,0.0001) # "flat" priors on GLM parameters}

for(h in 1:4){

gamma[h] ~ dnorm(0,0.0001) # "flat" priors on GLM parameters}

## Prior for random effect terms

for(i in 1:nID){

for(j in 1:1){

phi.mu[i,j] ~ dnorm(0,tau.mu[j]) }

phi.sd[i] ~ dnorm(0,tau.sd) }

## Random effect variances

tau.mu[1] ~ dgamma(1.5,37.5) #dgamma, mean model random intercept

sd.mu[1] <- abs(redun.mu[1])/sqrt(tau.mu[1]) # the implied prior is half t(0,5) with 3 degrees of freedom

#tau.mu[1] ~ dgamma(1.5,37.5) #dgamma, mean model random slope

#sd.mu[1] <- abs(redun.mu[1])/sqrt(tau.mu[1]) # the implied prior is half t(0,5) with 3 degrees of freedom

tau.sd ~ dgamma(1.5,37.5) #dgamma, sd model random intercept

sd.sd <- abs(redun.sd)/sqrt(tau.sd) # the implied prior is half t(0,5) with 3 degrees of freedom}

**Part 2**

library(rjags)

#Mean and SD of continuous variables

sqY<-data$Y.dist_Mass^2

sqX<-data$X.dist_Mass^2

meanuse = apply(cbind(sqY,sqX, data$RWD,data$Avg..raps),2,mean)

sduse = apply(cbind(sqY,sqX, data$RWD,data$Avg..raps),2,sd)

#library(car)

#leveneTest(sqy,Out)

#leveneTest(sqx,Out)

hist(data$Y.dist_Mass)

hist(sqY)

#Standardised continuous variables

StsqY = (sqY-meanuse[1])/sduse[1]

StsqX = (sqX - meanuse[2])/sduse[2]

StRWD = (data$RWD - meanuse[3])/sduse[3]

StAvgRaps = (data$Avg..raps-meanuse[4]/sduse[4])

#Grouping variables

Raps = data$No..raps

Out = data$Outcome

#ID and standardised observation number

ID = as.factor(data$Fight.ID) # individual index/Anemone ID

#use this for a nested analysis - this makes more sense use this

#POI = as.numeric(data$POI.no) # observation no. index

#StPOI = (POI - mean(POI))/sd(POI)

#--------------------code above this line loads the data & defines variables

### specify the setup for the MCMC

nchain = 3 # number of markov chains to run

nadapt = 1000 # burn-in

nrun = 20000 #20000 # posterior sample for each chain

#n.thin=100

### find the inital values

inituse = vector('list',nchain)

for(i in 1:nchain){

beta = rnorm(4) # initial value for GLM of mean

gamma = rnorm(4) # initial value for GLM for st. dev

tau.mu = rlnorm(1) # initial value for random effects precision parameters

tau.sd = rlnorm(1) # initial value for random effects precision parameters

redun.mu = rnorm(1) # initial value for redundant parameterization -- these should not be started at 0

redun.sd = rnorm(1) # initial value for redundant parameterization -- these should not be started at 0

inituse[[i]] = list(beta=beta,gamma=gamma,tau.mu=tau.mu,tau.sd=tau.sd,redun.mu = redun.mu,redun.sd=redun.sd) # list with all initial values

}

#--------------------have to have the jags model specified and saved as a .bug flie before next bit

# The following line runs the adaptive phase of the MCMC in JAGS

#run this model for random slopes on treatment.

jags.data =

list(y = StsqY,

Outx=1*(Out=="Ev"),

StRWDx=StRWD,

StAvgRapsx=SAvgtRaps,

IDx=ID,

nID=length(unique(ID)),

n=length(StsqY))

m1 = jags.model('BUGS_test skill_2_Evictions Only.R',

data = jags.data,inits=inituse,

n.chains = nchain, n.adapt = nadapt)

# The following line then finds the posterior sample for parameters listed

output = jags.samples(m1,c("beta","gamma", "sd.mu","sd.sd","re.mu","re.sd"), n.iter = nrun)

###Model checking

#coda.samples has to be used to get some of these metrics

output.a = coda.samples(m1,c("beta","gamma", "sd.mu","sd.sd","re.mu","re.sd"), n.iter = nrun)

#Convergence test - should be =<1.1 if more, increase no. interations

gelman.diag(as.mcmc.list(output$beta))

gelman.diag(as.mcmc.list(output$gamma))

gelman.diag(as.mcmc.list(output$sd.mu))

gelman.diag(as.mcmc.list(output$sd.sd))

gelman.diag(as.mcmc.list(output$re.mu))

#Autocorrelation

AC.1<-autocorr(output.a, c(0, 1, 5, 10, 50), relative=TRUE)

AC.1

#Effective sample sizes

ESN.1<-effectiveSize(output.a)

ESN.1

ESNmin<-min(ESN.1)

ESNmax<-max(ESN.1)

ESNminmax<-c(ESNmin,ESNmax)

names(ESNminmax) = c("Min", "Max")

ESNminmax

##### Summaries using jags.samples #####

### Parameters describing the mean model:

summary(as.mcmc(t(output$beta[,,1])))

### Parameters describing the standard deviation model:

summary(as.mcmc(t(output$gamma[,,1])))

### Parameters describing the random effects distributions

summary(as.mcmc(t(output$sd.mu[,,1])))

summary(as.mcmc(output$sd.sd[,,1]))

#######Psuedo-P

#Psuedo p-values (fixed effects)

tmp1 <- apply(output[["beta"]],1,function(M) mean(M>0))

tmp2 <- apply(output[["beta"]],1,function(M) mean(M<0))

p.beta <- 2*pmin(tmp1,tmp2)

p.beta

#Psuedo P for Gamma (SD model fixed effects)

tmp1 <- apply(output[["gamma"]],1,function(M) mean(M>0))

tmp2 <- apply(output[["gamma"]],1,function(M) mean(M<0))

p.gamma <- 2*pmin(tmp1,tmp2)

p.gamma
